# Effect of the Addition of Carbon Nanomaterials on Electrical and Mechanical Properties of Wood Plastic Composites

**DOI:** 10.3390/polym9110620

**Published:** 2017-11-16

**Authors:** Xingli Zhang, Xiaolong Hao, Jianxiu Hao, Qingwen Wang

**Affiliations:** 1 Key Laboratory of Bio-based Material Science and Technology (Ministry of Education), College of Material Science and Engineering, Northeast Forestry University, Harbin 150040, China; zhang-xingli@nefu.edu.cn (X.Z.); haoxiaolong.nefu@hotmail.com (X.H.); axiu518@126.com (J.H.); 2 College of Materials and Energy, South China Agricultural University, Guangzhou 510642, China

**Keywords:** wood plastic composites, carbon nanomaterial, electrical property, mechanical property

## Abstract

Wood Plastic Composites (WPCs) are a new generation of green composites that could optimize the use of harvested trees and increase the entire value chain. In this study, the electrical and mechanical properties of WPCs containing carbon blacks (CB), flake graphite (FG) and carbon nanotubes (CNTs) have been investigated. The electrical property of WPCs is improved significantly owing to the introduction of these carbon nanomaterial fillers. The volume and surface resistivity values of the investigated composites all obviously decreased with the increase in filler content, especially CNTs, which displayed the most satisfactory results. Based on a series of laboratory experiments carried out to investigate the mechanical performance, it can be concluded that the addition of the carbon nanomaterial fillers decreases the mechanical properties of WPCs slightly with the increase in filler content because of the weak interfacial interactions between the fillers and polymer matrix.

## 1. Introduction

Wood plastic composites (WPCs) have been widely utilized in siding, roofing, windows, door frames, and the outdoor furniture industry in recent years due to their improved mechanical properties, low processing cost, and biological performance. WPCs consist of varying contents of wood, plastics and additives, which are processed by thermoplastic shaping techniques such as extrusion, injection molding and compression molding [1,2,3]. WPCs show excellent electronic insulation properties; thus, WPC slats easily attract dust, which can cause static powder explosions and generate serious damage in equipment rooms, operating rooms or electronic component workshops [4]. To avoid these problems, antistatic WPCs must be developed by adding conductive filler to the polymer matrices during fabrication.

In terms of improving the electrical and mechanical properties of WPCs, the addition of conductive fillers is regarded as one of the most feasible methods, in view of its potential to intrinsically dispel static charge and confer dramatically improved electrical conductivity upon the host composites. Generally speaking, any conducting material (including metal oxides, metal powders, and carbon nanomaterials) can be used as conductive additives [5,6]. Compared to these conductive additives, carbon nanomaterials have superior properties, such as low weight, high chemical inertia and high specific surface area. It has been determined that carbon nanomaterials such as carbon blacks (CB), flake graphite (FG) and carbon nanotubes (CNTs) could be used as nano-fillers in polymer matrices for improving tensile strength, tensile modulus and electrical conductivity properties. Due to the percolation transition in the formation of conductive networks, using CB as conductive filler has been shown to offer significantly improved electrical conductivity for polypropylene (PP)/epoxy/glass fiber composites [7,8]. FG with low density, high electrical conductivity and great process ability, is suitable for use as an excellent conductive additive. Heo et al. [9] measured the electrical conductivity and flexural strength of conductive polymer composites reinforced with flak graphite particles, which were higher than the values for sphere-type particles due to the difference in the densification characteristic. CNTs have become an ideal reinforcing agent in a number of applications over the last decade, acting as electron acceptors and improving the dissociation of excitons by providing an enhanced electric field at the CNTs/polymer interface [10]. The variation of many parameters, such as CNT type, growth method and chemical pre-treatment, as well as polymer type and processing strategy, have given some encouraging results in fabricating relatively strong CNT-polymer composites [11,12,13,14,15]. However, these carbon nanomaterials, used as conductive fillers to enhance the electrical and mechanical properties of WPCs, have never been truly investigated.

In this research, we introduced CB, FG and CNT particles as conductive fillers to improve the electrical properties of WPCs. Particular emphasis was focused on assessing the effect of filler weight fractions on the morphology, and the electrical and mechanical properties of the WPCs.

## 2. Experimental 

### 2.1. Materials

Poplar wood fiber (WF) with particle sizes of 180 μm was used after being dried at 103 °C for 24 h (Baiquan, China). Polyethylene (PE) was obtained from Petrifaction Company (Daqing, China). PE had a density of 0.95 g/cm^3^ and a melt flow rate of 0.90 g/10 min. Conductive CB was purchased from Ouman Chemical (Shanghai, China) with a specific surface area of 900 m^2^/g. The mean diameter of the primary CB particles was around 35 nm, and the density at room temperature was 2.13 g/cm^3^. FG with an average particle size of 32–100 mesh and purity of 90–95%, was supplied by Tengshengda carbon Company (Qingdao, China). Multi-walled carbon nanotubes (MWCNTs) (Chengdu Institute of Organic Chemistry, China Co., Ltd., Chengdu, China), with purity >95%, length 10–30 µm, outer diameter: 10–20 nm, inner diameter: 5–10 nm, were used in this study. The carbon nanomaterials were used for conductive filler as received, without any further modification.

### 2.2. Sample Preparation

WF, PE, carbon nanomaterials and other additives, including maleic anhydride-grafted polyethylene (MAPE), coupling agent and lubricant, were mixed together in a high-speed mixer for 10 min, and subsequently extruded with a twin-screw WPC one-step extruder system (model: BHMS-40/75 WPC extruder, Nanjing Xawax Science Technology Co., Ltd., Nanjing, China) to directly produce WPC sheets (thickness was 4 mm and width was 100 mm). During extrusion, the temperatures of the melting zones, the pumping zone and the die zone were set to 160, 170 and 175 °C, respectively. All the composite specimens were prepared with the same processing conditions. For each researched system, the weight ratio of WF to PE for each of the experimental groups was around 6:4, the detailed material compositions of the WPCs are shown in Table 1.

### 2.3. Characterization

#### 2.3.1. Scanning Electron Microscopy

The morphology of the composites was observed by a scanning electron microscope (SEM, Quanta 200, FEI Company, Eindhoven, The Netherlands) instrument with an acceleration voltage of 12.5 kV. The specimens were cryogenically fractured in liquid nitrogen, and all of the fractured surfaces were coated with gold to enhance the image resolution and to prevent electrostatic charging. Transmission electron microscopy (TEM) observations of carbon nanomaterials were performed on a LEO-912AB transmission electron microscope (Carl Zeiss SMT AG Oberckochen, Munster, Germany) at an accelerating voltage of 100 kV.

#### 2.3.2. Electrical Properties

The volume resistance (*R_v_*) and surface resistance (*R_s_*) were measured by resistance meter GEST-121 (Beijing, China) at room temperature. The dimension of each sample used for the resistivity measurement was 4 mm × 100 mm × 100 mm. The volume resistivity ($\rho_{v}$) and surface resistivity ($\rho_{s}$) were determined using the following equation:

(1) $$\rho_{v}=R_{v}\frac{A}{h}$$

(2) $$\rho_{s}=R_{s}\frac{P}{g}$$

where *A* is the effective area of the guard electrode (*A* = 23.75 cm^2^); *h* is the average thickness of the samples; *P* is the effective perimeter of the guard electrode (*P* = 17.27 cm); *g* is the distance between the two electrodes (*g* = 0.5 cm). 

#### 2.3.3. Mechanical Properties

Tensile tests were carried out with a universal mechanical machine (Shenzhen Regear Instrument Cooperation, Shenzhen, China) according to ASTM D638-2004, with a sample size of 80 mm × 13.7 mm × 4 mm. The samples, measuring 80 mm × 10 mm × 4 mm, were used to test the impact strength in the un-notched mode according to ASTM D6110, using an impact tester (JC-5; Chengde Jingmi Testing Machine Co., Ltd., Chengde, China). Six replicates were tested for each sample group, and the averages were used for the comparison.

## 3. Results and Discussion 

### 3.1. Electrical Properties

**Figure 1:**
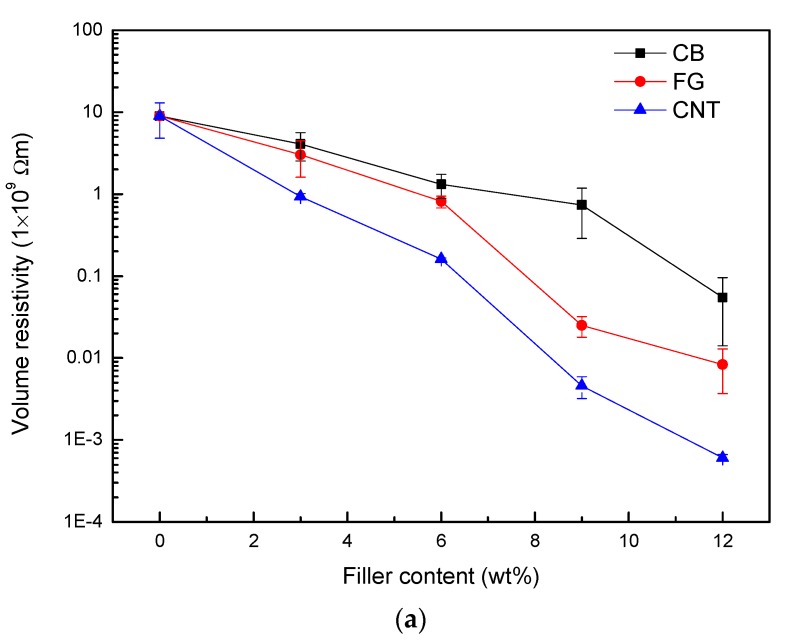

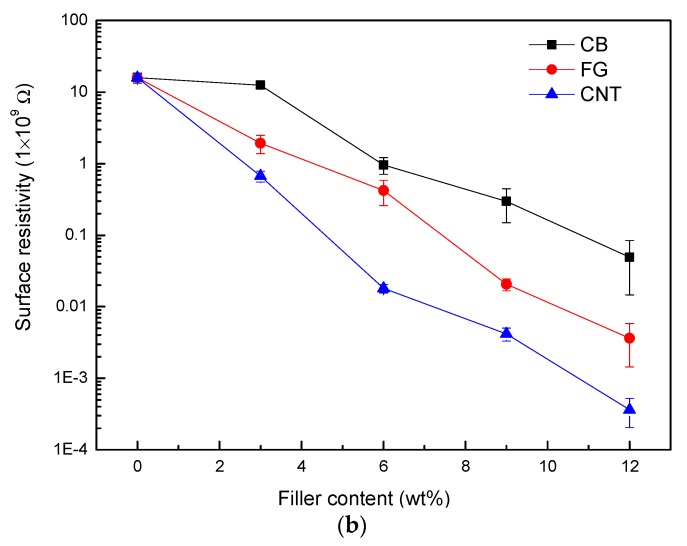
Electrical resistivity of WPC samples with respect to conductive filler content. (**a**) Volume resistivity; (**b**) Surface resistivity.

The volume and surface and resistivity values of the investigated composites are shown as a function of carbon nanomaterial content in Figure 1. The electrical resistivity of the WPCs decreased with an increase in carbon nanomaterial content. The decrease in electrical resistivity with the addition of carbon nanomaterials is due to the formation of a conductive network. With the same amount of carbon nanomaterial addition, the improvement in electrical conductivity (decreases in electrical resistivity) of the WPCs varies with the type of carbon nanomaterial used. CNTs have the best electrical conductivity, and CB has a relatively low electrical conductivity. For example, the surface resistivity of WPCs when adding 3 wt % CNTs is decreased by one order of magnitude in relation to that of pure WPCs. Remarkably, WPCs incorporating 12 wt % CNTs have a minimum volume resistivity of 6 × 10^5^ Ω·m. Electrical conductivities of neat polymer composite-incorporating CNTs have shown higher values than those measured in this work, mainly because the presence of wood fibers obstructs the construction of conductive networks [16]. Compared to CNTs, the addition of 3% CB particles did not significantly affect the electrical resistivity of WPC. This phenomenon has also been observed in PMMA/CB composites [17]. CNT-reinforced WPCs show better electrical conductively than that of CB, because of the high length-diameter ratio of CNTs, and their good innate electrical properties [18]. In addition, FG platelets with large surface area can easily form conductive pathways directly in the polymer matrix, so FG platelet-modified WPCs exhibit a significantly lower electrical resistivity in small weight fractions compared to the CB particles. 

### 3.2. Mechanical Poperties

**Figure 2:**
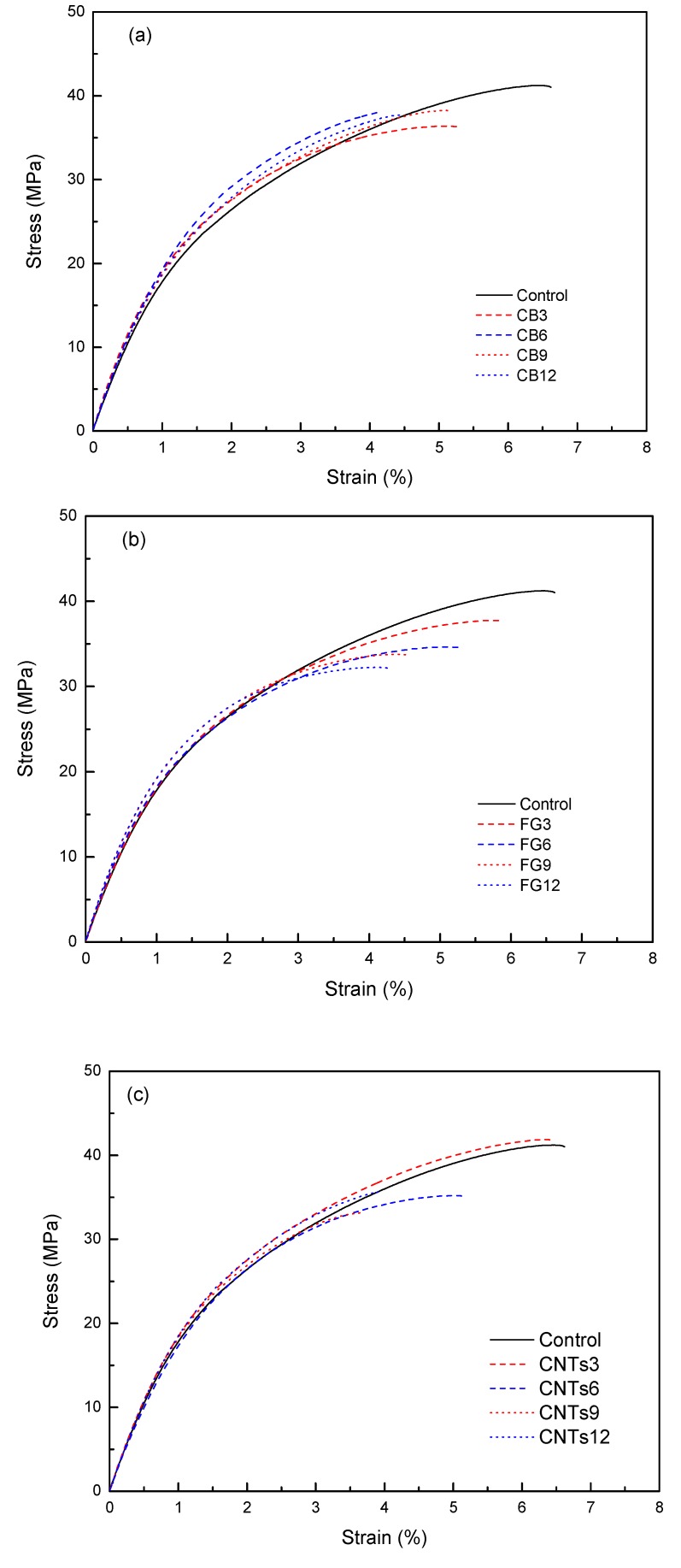
Tensile stress–strain curves of the composites. (**a**) CB; (**b**) FG; (**c**) CNTs.

**Figure 3:**
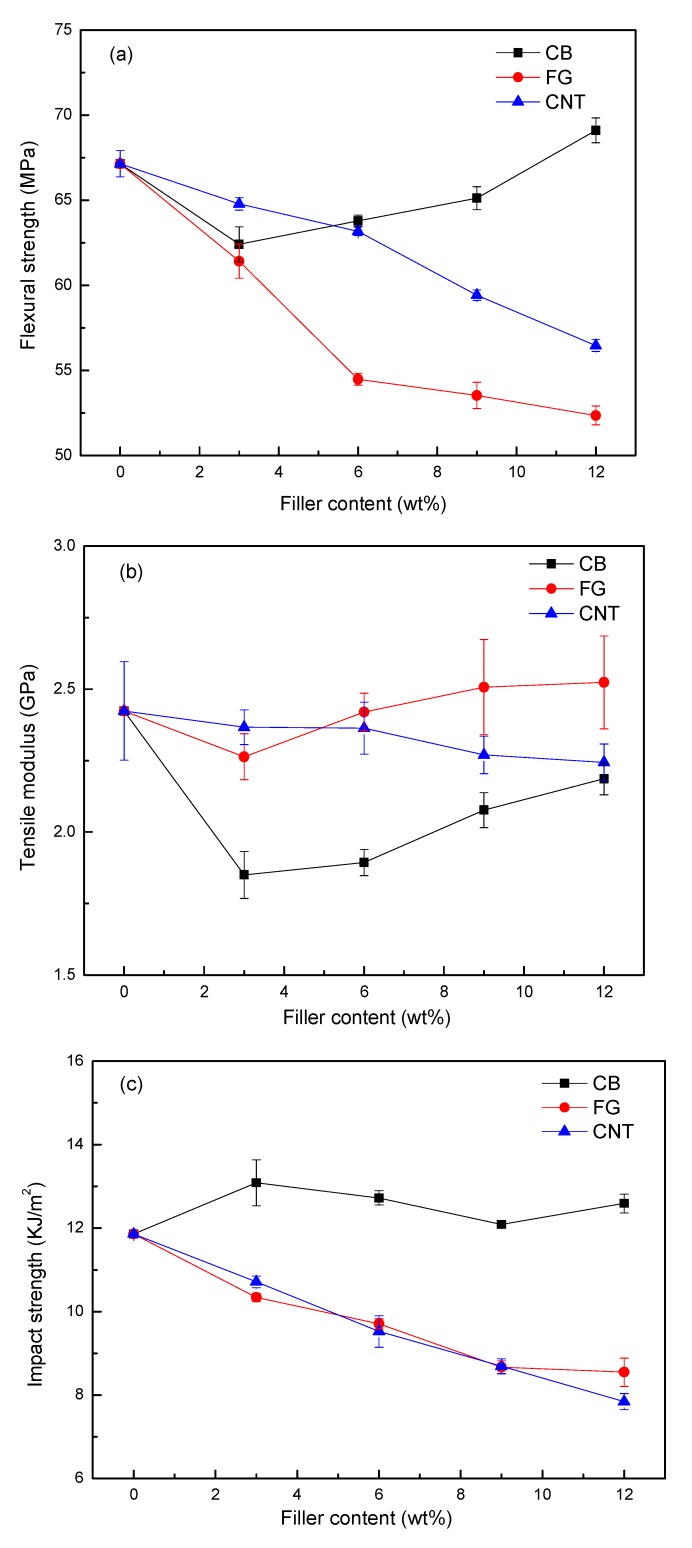
Mechanical properties of WPC samples with respect to conductive filler content. (**a**) Flexural strength; (**b**) Tensile modulus; (**c**) Izod impact strength.

Tensile tests were conducted to examine how the addition of various amounts of carbon nanomaterials influenced the mechanical properties of WPCs. Figure 2 illustrates the stress–strain curves of WPCs containing various amounts of carbon nanomaterials, while Figure 3 presents data showing the effect of the weight fraction of the filler on the flexural strength, tensile modulus, and izod impact strength of the WPCs. For CB particle-modified WPCs, no significant influence of the amount of nanoparticles in the flexural strength and tensile modulus was found (Figure 3a,b), but an increase in izod impact strength could be observed (Figure 3c) due to the interfacial interaction between the CB particles and the PE matrix [19]. The flexural strength, tensile modulus and izod impact strength of WPCs with higher CNT content decreased significantly, exhibiting a different trend from that of electrical conductivity. CNTs have shown great potential for increasing tensile strength [20]; however, our results contradict this theory, as the successive addition of CNTs to the composite leads to a tendency for aggregation. The aggregates of CNTs in the polymer matrix may counteract any enhancements that are weakly linked, and may act as defects in the composite. When the content is increased, the filler particles tend to form larger aggregates. Thus, the reinforcement effect of fillers at higher concentrations is suppressed by the weakening effect of the large aggregates. For FG particle-modified WPCs, the flexural strength and izod impact strength decreased significantly, while the tensile modulus increased, when FG fillers were added in an amount greater than 3 wt %. The presence of FG fillers in WPCs plays a significant role in improving the strength of WPCs, but the structural defects of the WPCs lead to a heterogeneous distribution of the stress, and thus to a decline in the stiffness. The tensile properties depend largely on the interfacial interaction in the composites [21].

### 3.3. Morphological Properties

**Figure 4:**
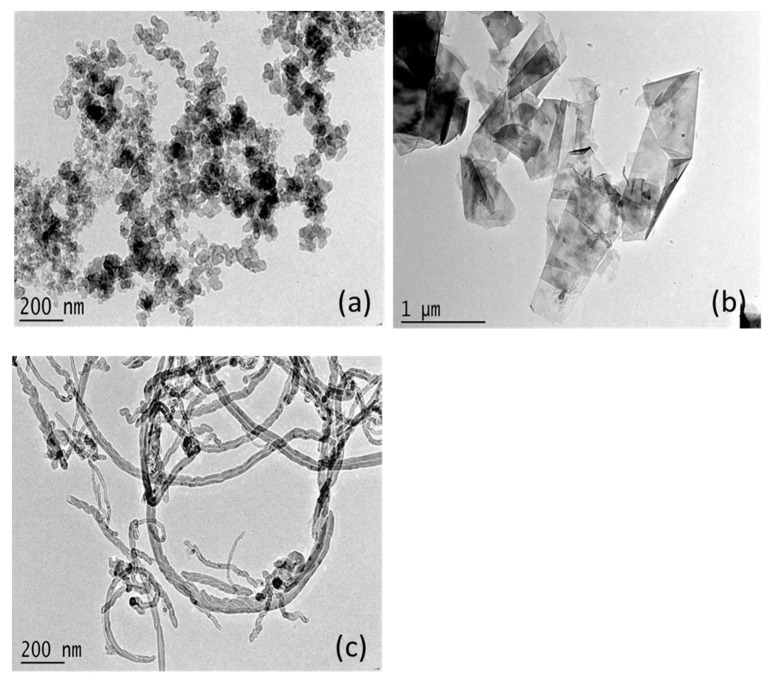
TEM micrograph of carbon nanomaterials. (**a**) CB; (**b**) FB; (**c**) CNTs.

Figure 4 shows the TEM image of the carbon nanomaterials. The CB particles exhibit a chain or grape-like structure, which would facilitate the formation of conductive networks in the polymer matrix. The original FG has a large surface area, which would improve its interactions with the polymer matrix. CNTs have a very high length–diameter ratio (small diameter, high length), so they are optimal for mechanical and electrical properties, but are difficult to disperse homogeneously in the matrix.

**Figure 5:**
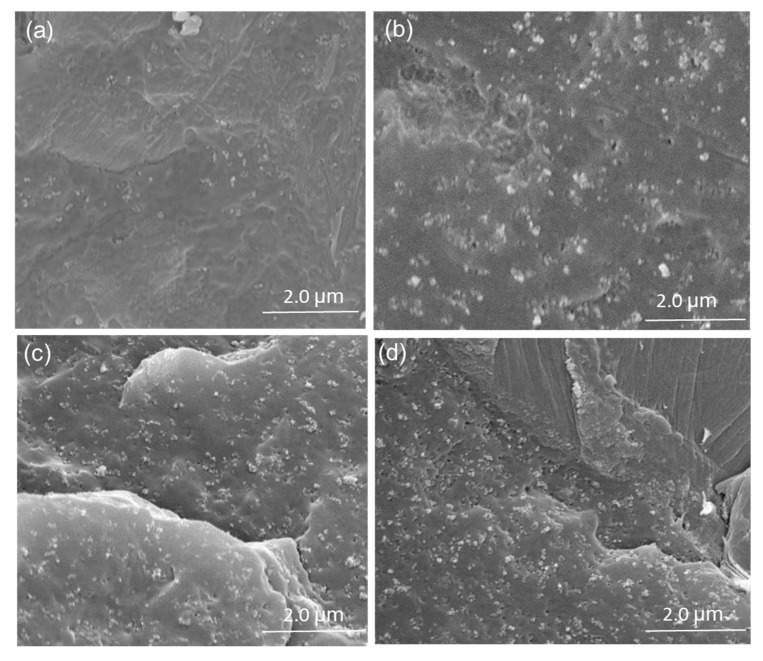
SEM micrographs of WPCs adding CB particles (**a**) 3 wt %; (**b**) 6 wt %; (**c**) 9 wt %; (**d**) 12 wt %.

The effective utilization of carbon nanomaterials in WPCs depends on their ability to disperse individually and homogeneously within the matrix material. Figure 5 shows the distribution of CB particles in WPCs, investigated by means of SEM. As shown in the micrographs, the composites prepared with the addition of CB exhibited a more uniform distribution of the nano-fillers. The average distance between CB particles in WPCs with 3 wt % CB added was about 1 µm, which was not able to form an electrically conductive path and effectively dissipate electronic charge. When the amount of CB added reached to 6 wt %, the average distance between CB particles was about 0.5 μm and conductive pathways were directly formed between CB particles [22,23]. This phenomenon is consistent with electrical resistivity results suggesting that small amounts of CB particles added to WPCs may not significantly enhance the electrical properties of WPCs.

**Figure 6:**
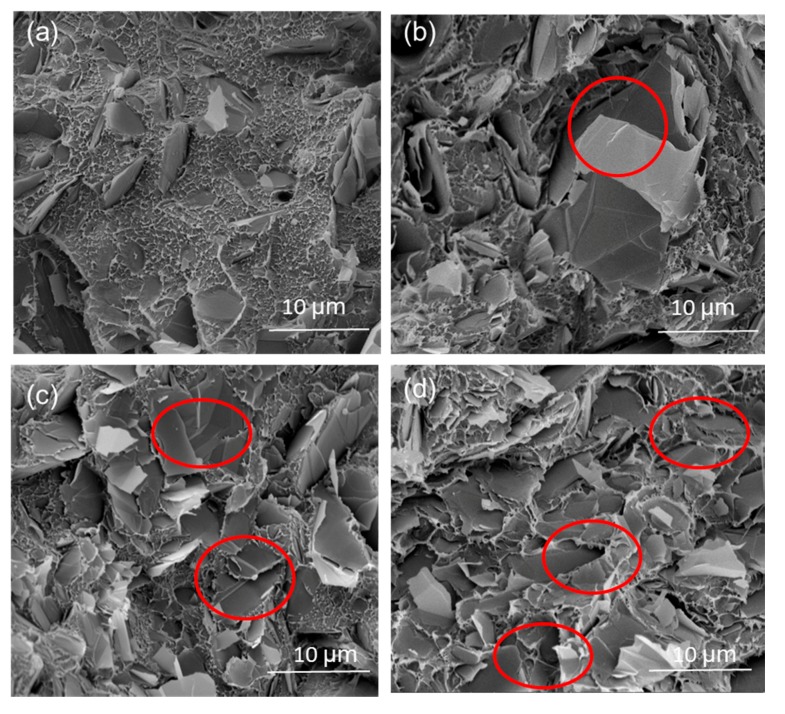
SEM micrographs of WPCs adding FG particles (**a**) 3 wt %; (**b**) 6 wt %; (**c**) 9 wt %; (**d**) 12 wt%.

From Figure 6, it can be observed that the FG platelets in the WPCs at lower filler weight fractions disperse discretely in the PE matrix, which may result in a lower value for the electrical conductivity of the composites. FG at high weight fractions formed an interconnecting network in the immiscible polymer blends, thus contributing to the rapid increase in the electrical conductivity of the system. The SEM images also show that FG platelets begin to agglomerate with a further increase in content, as indicated by the circle in Figure 6. The particles interrupt the generation of cross-linked bonds in the composite, which contributes to the decline of mechanical properties. 

**Figure 7:**
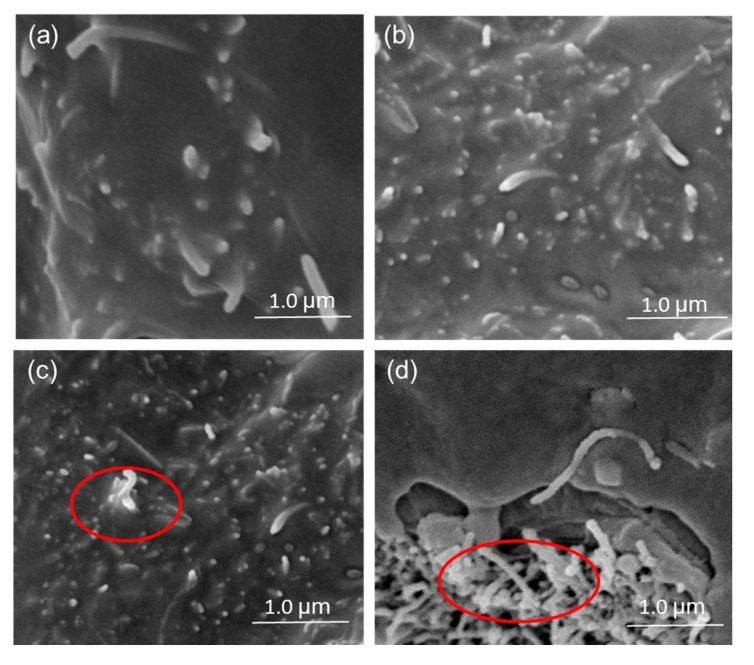
SEM micrographs of WPCs adding CNTs particles (**a**) 3 wt %; (**b**) 6 wt %; (**c**) 9 wt %; (**d**) 12 wt %.

The MWCNT particles are tightly covered by the PE, and are well dispersed when the content of MWCNTs is 3 wt % (Figure 7a). Similar to the FG platelet modification, it is possible to find MWCNT clusters with the increase of MWCNT content in the PE matrix. Although MWCNTs have good potential for increasing electrical and mechanical properties, undesired remnants from the production process and MWCNT agglomerates act as flaws in the PE matrix, and oppose the desired enhancement.

All in all, the CBs were well dispersed, and no significant clusters or agglomerates were found; however, the dispersion and purity of the FG and CNT particles should be considered carefully, as they may make any improvements in the properties of WPCs less obvious. 

## 4. Conclusions 

In this study, WPCs were prepared by incorporating carbon nanomaterials (carbon blacks, flake graphite and carbon nanotubes) to improve their electrical and mechanical properties. Meanwhile, the electrical and mechanical properties of the modified WPCs were also investigated.

The electrical resistivity of WPCs decreased with the increase of carbon nanomaterial content, which indicates that increasing the number of carbon nanomaterials provides greater opportunity to form an interconnected conductive network. The mechanical property of WPCs when adding carbon nanomaterial fillers decreased slightly with the increase of filler content. SEM observation shows that the CBs were well dispersed, but it was possible to find FG and CNT clusters or agglomerates with an increase in the weight fraction in the PE matrix. In other words, the electrical and mechanical properties of WPCs modified by carbon nanomaterials showed opposite tendencies, indicating that we need to seek a balance between these two types of properties.

## Figures and Tables

**Table 1:** Material compositions of the WPCs.

Sample	WF (wt %)	PE (wt %)	CB (wt %)	FG (wt %)	CNTs (wt %)
Control	60	40	0	0	0
CB3	58	39	3	0	0
CB6	56	38	6	0	0
CB9	54	37	9	0	0
CB12	52	36	12	0	0
FG3	58	39	0	3	0
FG6	56	38	0	6	0
FG9	54	37	0	9	0
FG12	52	36	0	12	0
CNTs3	58	39	0	0	3
CNTs6	56	38	0	0	6
CNTs9	54	37	0	0	9
CNTs12	52	36	0	0	12
